# Cardiometabolic Risk Factors in Pregnancy and Implications for Long-Term Health: Identifying the Research Priorities for Low-Resource Settings

**DOI:** 10.3389/fcvm.2020.00040

**Published:** 2020-03-20

**Authors:** Shobhana Nagraj, Stephen H. Kennedy, Robyn Norton, Vivekananda Jha, Devarsetty Praveen, Lisa Hinton, Jane E. Hirst

**Affiliations:** ^1^Nuffield Department of Women's and Reproductive Health, University of Oxford, Oxford, United Kingdom; ^2^The George Institute for Global Health, Oxford, United Kingdom; ^3^The George Institute for Global Health, University of New South Wales, Sydney, NSW, Australia; ^4^The George Institute for Global Health, New Delhi, India; ^5^Manipal Academy of Higher Education, Manipal, India; ^6^The Nuffield Department of Primary Care Health Sciences, University of Oxford, Oxford, United Kingdom; ^7^THIS Institute (The Healthcare Improvement Studies Institute), University of Cambridge, Cambridge, United Kingdom

**Keywords:** high-risk pregnancy, preeclampsia, gestational diabetes, cardiometabolic disorders, cardiovascular disease

## Abstract

Cardiometabolic disorders (CMDs), including ischemic heart disease, stroke and type 2 diabetes are the leading causes of mortality and morbidity in women worldwide. The burden of CMDs falls disproportionately on low and middle-income countries (LMICs), placing substantial demands on already pressured health systems. Cardiometabolic disorders may present up to a decade earlier in some LMIC settings, and are associated with high-case fatality rates. Early identification and ongoing postpartum follow-up of women with pregnancy complications such as hypertensive disorders of pregnancy (HDPs), and gestational diabetes mellitus (GDM) may offer opportunities for prevention, or help delay onset of CMDs. This mini-review paper presents an overview of the key challenges faced in the early identification, referral and management of pregnant women at increased risk of CMDs, in low-resource settings worldwide. Evidence-based strategies, including novel diagnostics, technology and innovations for early detection, screening and management for pregnant women at high-risk of CMDs are presented. The review highlights the key research priorities for addressing cardiometabolic risk in pregnancy in low-resource settings.

## Introduction

Significant physiological changes occur during pregnancy. These may affect a woman's cardiovascular, immune and metabolic functions and unmask susceptibility for developing cardiometabolic disorders (CMDs) (1, 2). Women with a history of Hypertensive Disorders of Pregnancy (HDPs, including preeclampsia), Gestational Diabetes Mellitus (GDM), spontaneous preterm birth, and delivery of a small for gestational age (SGA) baby, display increased risk of future CMDs: including cardiovascular disease (CVD), stroke, Type 2 Diabetes (T2DM) and chronic kidney disease (CKD) (3–7). Pregnancy complications are no longer seen as isolated conditions affecting pregnancy, but independent risk factors for future CVD (5, 8).

Women are more likely to die from CVD because of late presentation and differences in symptomatology (9). The burden of premature deaths from complications of pregnancy such as preeclampsia, preterm birth and SGA babies and CVD later in life, fall disproportionately upon Low-and Middle-Income Countries (LMICs) (10, 11). Women in rural areas of LMICs are further disadvantaged due to limitations in healthcare access and infrastructure, issues of poverty, and educational and socio-cultural barriers in accessing timely care as well as engaging with ongoing treatment (12, 13).

This review discusses the research priorities for improving women's cardiometabolic health in low-resource settings in LMICs following a high-risk pregnancy. We focus on three main areas related to the provision of universal health coverage: (1) Community-level interventions for high-risk pregnancies; (2) The need for life-course based approaches to women's health, and (3) Improving equity and access to affordable treatments for CMDs.

### Community-Level Interventions for High-Risk Pregnancies

Preventative efforts to avert CMDs often start too late to be effective (3). Early detection and management of women with pregnancy complications associated with a high-risk of future CMDs requires active community engagement to encourage early, regular antenatal care (ANC) attendance (14).

In low-resource settings, where multiple clinic visits may not be feasible, point-of-care tests (POCT) have been used by Community Health Workers (CHWs), nurses and doctors to screen for HIV/AIDS, malaria and anemia during pregnancy (14). Potential biomarkers for first trimester preeclampsia screening include serum placental growth factor (PlGF), serum pregnancy-associated plasma protein A (PAPP-A), mean arterial pressure (MAP) and uterine artery pulsatility index (UTPI) (15). Point of care tests for these emerging biomarkers merit further country-specific, clinical, and economic evaluation (16). The implementation of novel diagnostics in LMICs, must be coupled with upgrading of laboratory facilities in primary care settings, which are insufficiently prioritized by governments worldwide (17).

The detection of HDPs mainly involves blood pressure (BP) measurement and urinalysis. Whilst non-invasive, these skills can be intimidating to rural healthcare workers. To improve community-level detection of HDPs in LMICs, low-cost instruments have been developed and validated for use by frontline healthcare workers in low-resource settings. The CRADLE Vital Signs Alert device is a novel, semi-automated BP device, validated for use in pregnancy and to stratify risk in pregnant women with both high and very low BP, costing ~$20 USD (18). Feasibility studies have shown high levels of acceptability by Community Health Workers (CHWs) in Nigeria, Mozambique, Zimbabwe, Ethiopia and India (18, 19). A large stepped-wedge cluster-randomized controlled trial across 10 LMICs was; however, unable to demonstrate impact on the primary composite outcomes of maternal mortality and morbidity (20). This may be due, in part, to insufficient power and sample size, and significant variations between clusters (20). Low cost urinalysis devices for proteinuria detection have also been developed and piloted (21–23), although evidence of impact on clinical endpoints is lacking.

Community-based screening for GDM is complicated by a lack of attendance, and lack of consolidated criteria for testing and diagnosis of GDM (24). Routine application of the gold standard fasting oral glucose tolerance test (OGTT), followed by venous blood being drawn at 0, 1, and 2 hours post-glucose load is not feasible in rural settings where mothers have to travel long distances and wait substantial amounts of time to receive antenatal services, and healthcare staff skilled in drawing blood are not available at specified times (24). Some rural centers perform OGTTs irrespective of fasting status. A study from India, testing women irrespective of their fasting state, did not reveal statistically different results compared to the WHO-recommended fasting 75-g OGTT (25). Subsequent studies have, however, found the sensitivity of non-fasting tests to be low (26). In response to the growing burden of GDM in India, pragmatic guidelines for low-resource settings have been developed (24, 27); however, the operability of such guidelines relies heavily on the presence of good laboratory and primary care infrastructure.

### The Role of Mobile Technologies

Mobile health (mHealth) technologies have the potential to increase equity, quality and efficiency of service delivery in LMICs (28). mHealth technologies have contributed to reductions in delays in accessing maternal health in LMICs (29), and can be useful in the diagnosis, monitoring, providing clinical decision support, education and health promotion (30, 31).

A large-scale cluster randomized trial of a multi-faceted smartphone-based mHealth intervention (ImTECHO) used by CHWs to deliver care to pregnant women in their homes, involving a population of almost half a million in rural Gujarat in India, demonstrated improved engagement and delivery of antenatal and postnatal care by CHWs (32). The platform facilitated longitudinal tracking, scheduling of health services, screening for complications, counseling and behavior change communication, and real-time mentoring and supportive supervision of CHWs. This study highlighted the feasibility and effectiveness of mobile phone technologies as job aids to frontline healthcare workers to strengthen the local health system, but did not demonstrate a positive impact on maternal or neonatal mortality (32).

Similar interventions have potential to extend beyond the immediate postpartum period for long-term follow up of women at high risk of future CMDs. Future research should focus on rigorous evaluation of mHealth interventions beyond pilot studies (33), and include process evaluation and cost-effectiveness analyses, with a focus on local ownership and integration within existing health systems.

Risk stratification tools for pregnant women with preeclampsia have been developed for predicting risk of adverse maternal outcomes (34, 35). The full-Pre-eclampsia Integrated Estimate of RiSk model (fullPIERS) is a prediction model based on clinical history, signs and symptoms and laboratory tests. Developed in a High-Income Country (HIC) context, it has also been validated for use in low-resource settings (36, 37); however, is reliant on full laboratory-based support (35). A succinct version, based on symptoms and signs alone (miniPIERS), has also been developed for community-based risk assessment (34). These tools provide clinical decision support to frontline healthcare workers and may be integrated into mHealth platforms, such as the PIERS-on-the-move (POM) mHealth platform (38). A study of the POM mHealth platform, demonstrated good levels of acceptability, feasibility, and moderate utility for the prediction of adverse maternal outcomes in women with HDPs (39).

While these tools may be used for identification and risk-stratification of high-risk women during pregnancy, little evidence is currently available to calculate or predict long-term cardiovascular risk in this population (40). Robust data collection systems are needed for the long-term follow-up of women in LMICs to study the true prevalence and impact of high-risk conditions in pregnancy on future CMDs, and enable accurate risk stratification of high-risk women. It is unclear if existing cardiovascular risk prediction models could be improved through the addition of history of pregnancy complications (41, 42). There is a need for prognostic models using sample populations reflecting the diversity of target populations, and involving both nulliparous and multiparous women to better identify women at high-risk of CMD during and after pregnancy (40). Women who develop T2DM following GDM in some LMIC settings are more likely to exhibit certain characteristics such as increased body mass index postpartum, family history of T2DM, and certain ethnicities (43, 44). It is unclear, however, if these clinical features may be used to guide risk stratification of women with GDM and their progression to T2DM across other LMIC settings, as they are based on small-scale studies. A systematic review on the progression of GDM to T2DM concluded that a markedly raised fasting glucose level during pregnancy was most highly predictive of progression to T2DM, and did not support the use of features such as ethnicity, BMI, and family history of T2DM for risk stratification of progression to T2DM in pregnant women (45).

### Task-Sharing in the Community

Task-sharing has potential to empower and engage community members, improve efficiency, and “*expand the reach of delegated medical acts”* (46). In areas with a shortage of doctors and nurses in LMICs, CHWs have been deployed to deliver interventions for the early detection of high-risk pregnant women (18, 47–49) and enable community-based data collection (50). CHWs have high levels of trust and respect within their communities, and motivate women to engage with antenatal care (51). Task-sharing relies upon continuous training and supervision, as CHWs may have limited literacy in low-resource settings (14).

The community-based management of hypertension in Nepal (COBIN) cluster randomized controlled trial in the general adult population of Nepal, established the effectiveness of a CHW-led home-based health education and screening for the reduction of Systolic BP (of almost 5 mmHg), in adults with hypertension; and amelioration of age-related increases in BP in adults without hypertension (52). Further examples of community-based programmes with potential to reduce cardiovascular risk in LMIC settings exist (53–56), however as the COBIN trial team concluded; long-term trials with hard clinical outcomes, such as myocardial infarction and stroke as primary endpoints are needed to confirm the effect of CHW-led interventions on cardiovascular mortality and morbidity (52). Important areas for future research would be to conduct adequately sized, robustly designed trials, demonstrating tangible impact upon mortality across the life-course, including cost effectiveness analyses, and exploration of the impact of climate change and seasonal variations on BP-related endpoints (57, 58).

### LMIC-Based Data Repositories and Biobanks

Research associating pregnancy complications with CVD risk have, to date, been derived from linkage of large national data sets from high income countries (HIC) (41, 59, 60). Unlike HICs, the majority of CVD deaths in sub-Saharan Africa are due to stroke rather than ischemic heart disease (61), which may reflect differences in etiology. Currently, there are insufficient data on the life-long health of women living in LMIC settings. Encouraging collaboration across LMICs to form consortia for uniform women's health related data collection such as the COLLECT database for collaborative pregnancy and placental research (62), started by the Global Pregnancy Collaboration (CoLab) (63), might facilitate the use of big data analytics to enable risk stratification of women with pregnancy-related risk factors for CMDs in LMICs, and identify key timings for interventions.

With the fast-developing world of genomics, proteomics and metabolomics, LMICs might benefit from establishing biobanks. This would encourage locally-driven -omics research, based on the needs and priorities of LMICs, with local data ownership. South-south as well as north-south collaborations have potential to improve research into biomarkers for risk factors for CMD in women, including pregnancy-related risk factors such as preterm birth, pre-eclampsia, and GDM. The significant genetic variations in South Asian and African populations are important to furthering our knowledge of disease etiology and drug development. Currently, the majority of DNA used for research studies come from participants of European descent, with only 2% of data contributed from African data sets (64). In response, a new pan-Africa biobank start-up, 54-gene (64) and additionally, the first pan-Asia biobank have launched (65), both with the aim of solving the problem of lack of global representation. Similar initiatives are found in Brazil (66). Due to the heterogeneity of the samples and different collection strategies of existing biobanks, adequate skills training, capacity-building of LMIC-based researchers, and regulatory environments would need to be in place to support standardization of biobanks globally (63), as well as the infrastructure (such as 24-h electricity) for sample storage.

## The Need for Life-Course Based Approaches to Women's Health

Health systems in low-resource settings are often designed to provide emergency services only. Preventative services, however, are fundamental to ensuring a healthy population. A recent study showed that each dollar spent on a package of essential preventive services leads to a net health gain of 1.8 dollars in India (67). Provision of integrated care for women throughout their life-course is one way in which women may be engaged within the health system at key intervals in their life. By using entry points (e.g., antenatal care) into the health system as opportunities to engage women, opportunistic screening for cardiometabolic risk factors might be feasible at critical points during the life-course.

There are few integrated care models that link antenatal care and non-communicable disease (NCD) prevention (68–71), although those demonstrating effectiveness for *communicable* diseases such as HIV and life-long health, exist (72, 73). Future research into how existing successful models of integrated care (such as the HIV programmes in sub-Saharan Africa) could be adapted for NCD prevention will be valuable in designing health systems responsive to the needs of women throughout their life-course.

There is a 2-fold increased risk of developing CVD, and a 3-5-fold risk of chronic hypertension in the decades following a pregnancy complicated by HDP (59, 74–77). The cumulative incidence of T2DM following GDM increases markedly within the first 5 years postpartum and plateaus after 10 years (45, 78). The American Heart Association (79) and the American Diabetes Association (80) have recommended incorporating pregnancy-related risk factors as part of screening for adult cardiovascular disease (81). Postpartum screening for ongoing problems with BP (79, 82) and glucose control through the use of an OGTT at 6–12 weeks postpartum and 1–3 yearly thereafter are advised (80). Despite these recommendations, most women with a complicated pregnancy do not routinely receive postpartum follow-up (83, 84) and certainly not for 5 years following the index pregnancy, when the long-term sequelae are likely to manifest.

Although challenges to postpartum screening of women are faced worldwide, there are specific contextual challenges in LMICs. Postnatal follow-up is lower in LMICs than in high income settings (85, 86). In low-resource settings, the burden of CMDs on daily life, household expenditure and economic stability have considerable implications for women and their entire household. Cultural practices after birth, workforce shortages, particularly in rural areas, and a lack of health system infrastructure are additional barriers to providing life-long care. Many women with hypertension and T2DM remain undiagnosed, although population-based screening for GDM shows high rates of conversion from GDM to T2DM in both urban and rural areas of LMICs (87). Further education and training of women and healthcare staff are needed to encourage postpartum follow-up and repeat testing of women at high risk of CMDs (84). Postpartum interventions targeting high-risk women might learn from adult NCD prevention programmes that have shown evidence of clinical benefit (88, 89). Successful lifestyle interventions are characterized by addressing more than one area of prevention and taking a holistic approach to change (90).

## Improving Equity and Access to Affordable Treatments for CMDs

A study of 596 urban and rural communities in 18 countries concluded that improving the availability and affordability of medicines for CMDs is essential for increasing their uptake and use (91). This is of great importance for women identified at high-risk of CMDs early in their life-course. Although common medications for cardiovascular disease and diabetes are widely available in some LMICs, the out-of-pocket expenditure for households already struggling to meet their daily needs, is a significant barrier to their continued life-long use for women diagnosed with CMDs earlier in their life-course. The situation is even more pronounced in rural areas (92). Even with improved access to affordable medicines, there are significant socio-cultural barriers affecting compliance to lifelong treatment (93).

The WHO has committed to achieving the goal of 80% availability of affordable, essential medicines for NCDs by 2025 in their Global Action Plan (94). Current rates of medicine use for the secondary prevention of CVD are, however, substantially lower (91, 92, 95). The proportion of patients with coronary heart disease receiving medications for secondary prevention of CVD in 10 countries (including several LMICs) in the Prevention of REcurrences of Myocardial Infarction and StrokE (WHO-PREMISE) PREMISE study, was lower than 50% for all major classes of CVD prevention medicines, including beta blockers (48%), ACE inhibitors (40%), and statins (30%) (96).

Governments need to set policy objectives to ensure that essential medicines to stem the tide of the rising CMD epidemic are affordable and available to their populations, including those in rural areas, by ensuring continuity of supply chains, i.e., the manufacturing, transport and distribution of medicines. Multidisciplinary research is needed to explore socio-cultural barriers to prescribing and taking medications, if we are to ensure that women with, or at risk of CMDs in LMICs, receive essential care.

## Discussion: The Research Agenda for LMICs

Only a multi-dimensional research strategy can help improve women's health in LMICs. Our review highlights the need for further well-designed experimental studies of novel technologies and biomarkers, embedded within the real-world context. Such studies would need to be adequately powered to demonstrate tangible benefit to clinical outcomes, such as maternal and neonatal mortality in the short-term, and cardiovascular endpoints in the longer term, and include cost-effectiveness analyses for future scalability. Data collection and monitoring are important strategies for improving healthcare provider practices (90). Future research should prioritize high-quality community-based data collection and linkage to existing hospital level health information systems, through prospective cohort studies with appropriate representation of women living in low-resource settings. Exploration of the complex social factors that impact the health of women both during pregnancy and beyond, with reference to CMDs has also been highlighted as a key area for future research (see Table 1).

**Table 1:** Summary of research recommendations.

**Recommended area of research**	**Sub-topics**	**Skills required**
Community-level interventions for High-Risk Pregnancies (HRPs)	Development and evaluation of affordable point of care tests for HRPs.	Community engagement for developing contextually-relevant and usable tools in low resource settings.
	Designing and evaluating community-based interventions through robust clinical trials and significant clinical endpoints.	Novel methodologies for real-world, pragmatic clinical trials.
	Pragmatic evidence-based guidelines for screening and diagnosis of HRPs in the community.	Contextual-based multidisciplinary research to guide development.
	Building the evidence-base for accurate cardiovascular risk prediction in high-risk pregnant women.	Building capacity for local LMIC biobanks and data repositories, alongside improving local research capabilities and governance systems.
	Task-sharing and workforce planning	Understanding the needs of healthcare workers in low- resource settings.
Life-course approaches to women's health	Integrated care linking antenatal care to long-term women's health.	Learning from other successful models for integrated care throughout the life-course e.g., HIV.
Improving equity and access	Advocacy aimed at Governments to provide essential medications for secondary prevention of CMDs.	
	Understanding the socio-cultural barriers to prescribing and medicine use in low resource settings.	Contextually-based research involving social scientists and anthropologists.

The Academy of Medical Sciences have emphasized the need to develop locally driven solutions and diagnostics for NCDs, including disruptive technologies (17). Significant challenges include the commercially unattractive nature of research into novel low-cost diagnostics for low-resource settings, affecting the development and scalability of new diagnostic tests (17). Nevertheless, given that pregnancy complications associated with future CMDs still result in significant maternal mortality worldwide (97), there is a moral imperative to give women in LMICs the same access as those living in HICs to screening tests that predict life-threatening conditions in pregnancy, and beyond.

## Author Contributions

SN was responsible for the conceptualisation of the mini-review and for writing the first draft. All authors contributed to the subsequent editing and review of the draft paper for publication.

### Conflict of Interest

The authors declare that the research was conducted in the absence of any commercial or financial relationships that could be construed as a potential conflict of interest.
